# Interventional Study of Nonpharmaceutical Measures to Prevent COVID-19 Aboard Cruise Ships

**DOI:** 10.3201/eid3005.231364

**Published:** 2024-05

**Authors:** Varvara A. Mouchtouri, Leonidas Kourentis, Lemonia Anagnostopoulos, Michalis Koureas, Maria Kyritsi, Katerina Maria Kontouli, Fani Kalala, Mattheos Speletas, Christos Hadjichristodoulou

**Affiliations:** European Union Healthy Sailing Project, Larissa, Greece (V.A. Mouchtouri, L. Kourentis, L. Anagnostopoulos, K.M. Kontouli, C. Hadjichristodoulou);; European Union Healthy Gateways Joint Action, Larissa (V.A. Mouchtouri, L. Kourentis, L. Anagnostopoulos, C. Hadjichristodoulou);; University of Thessaly, Larissa (V.A. Mouchtouri, M. Koureas, M. Kyritsi, F. Kalala, M. Speletas, C. Hadjichristodoulou)

**Keywords:** SARS-CoV-2, COVID-19, coronavirus, severe acute respiratory syndrome coronavirus 2, viruses, respiratory infections, zoonoses, antibodies, cruise, ship, vessel, quarantine, isolation, outbreak, investigation, mask, facemask, FFP2, N95, vaccine, physical distancing, surveillance, travel, sanitation, nonpharmaceutical measures, Europe

## Abstract

Cruise ships carrying COVID-19–vaccinated populations applied near-identical nonpharmaceutical measures during July–November 2021; passenger masking was not applied on 2 ships. Infection risk for masked passengers was 14.58 times lower than for unmasked passengers and 19.61 times lower than in the community. Unmasked passengers’ risk was slightly lower than community risk.

In the summer of 2021, several European Union Member States (EUMS) and European Economic Area (EEA) countries gradually lifted COVID-19 public health measures and reopened borders. The easing of restrictions enabled cruise lines to resume operations, applying guidelines published by the EU Healthy Gateways Joint Action, the European Centre for Disease Prevention and Control, and European Maritime Safety Agency. We assessed the effectiveness of nonpharmaceutical measures (NPMs) by comparing COVID-19 incidence rates among EUMS and EEA communities and populations of cruise ships and applying different sets of measures.

## The Study

We conducted an ecologic study in which cruise ships in group 1 (passenger and crew populations on 2 cruise ships, ships A and B) and group 2 (passenger and crew populations of 9 cruise ships) carrying vaccinated populations applied identical NPMs apart from face masking in passengers and physical distancing, which group 1 did not apply (*1*) (Table). The cruise ship company provided epidemiologic data and screening and diagnostic results for group 1 (Appendix). Ship captains or doctors reported epidemiologic data and screening and diagnostic results to competent health authorities and EU Healthy Gateways Joint Action (Appendix). Passenger populations changed in every cruise, but ≈6 passengers remained onboard the ship for >1 voyage. COVID-19 imposed severe crew change restrictions, and most crew remained the same during the study; the percentage of crew disembarking likely represented <0.5% of the crew population. We calculated COVID-19 incidence rates for the period of July–November 2021 for groups 1, 2, and 3 (EUMS communities). We obtained epidemiologic data for EUMS communities from the European Centre for Disease Prevention and Control website (*4*).

**Table:** COVID-19 health measures, laboratory screening, and diagnostic testing for SARS-CoV-2 per comparison population group in interventional study of nonpharmaceutical measures to prevent COVID-19 aboard cruise ships*

Variable	Comparison population groups		
	Group 1: cruise ships A and B sailing in EUMS waters	Group 2: 9 cruise ships sailing in EUMS waters	Group 3: EUMS/EEA community populations
Mask wearing	Unmasked passengers, masked crew†	Masked passengers and crew	Policies varied
Physical distancing‡	N	Y	Policies varied
Daily body temperature measurement for passengers and crew	Y	Y	NA
Pre-embarkation health screening questionnaire for passengers and crew§	Y	Y	NA
Quarantine measures for close contacts of SARS-CoV-2–positive passengers and crew members	Y	Y	Yes
Buffet line allowed in food service areas¶	Y	N	NA
>95% passengers and crew members vaccinated#	Y	Y	Vaccine coverage varied in EUMS
Serologic testing for crew members	Y	N	NA
End of voyage reporting by cruise line to competent authorities for COVID-19 surveillance data	Y	Y	NA
Other NPMs: education and training; restrictions for population density, excursions, and port visit; policy enforcement	Y	Y	Policies varied**
Screening/diagnostic testing for crew members			
All crew members already onboard the cruise ship tested by RADT within 1 wk before resuming operations	Y	Y	NA
Day of embarkation RADT	Y	Y	NA
Routine RADT	Every 7 d	Every 7 d	Varied among EUMS
Screening/diagnostic testing for passengers			
Day of embarkation RADT	Y	Y	NA
RADT before disembarkation	Y	Y	NA
Nonvaccinated (or not fully vaccinated) passengers tested by RADT on day 3 or 4 of cruise††	Y	Y	NA

*See Appendix (https://wwwnc.cdc.gov/EID/article/30/5/23-1364-App1.pdf) for more detailed information about definitions and methods used in the study. EEA, European Economic Area; EUMS, European Union member states; NA, not applicable; NPM, nonpharmaceutical measures; RADT, rapid antigen detection test. †All passengers wore masks on 1 voyage in which elevated number of cases occurred in cruise ship A. ‡Physical distancing of 1.5 m. §Information collected included demographic information (name, date/time of itinerary, port of disembarkation, cabin number, contact telephone number for 14 d after disembarkation), health questions regarding the past 14 d (presence of COVID-19 compatible symptoms, close contact of COVID-19 case, and whether person provided care was in close proximity, traveled on conveyance, or shared household with SARS-CoV-2–positive person). ¶Group 1 ships provided meals as sitting service and in a buffet line with strict hand hygiene measures, sneeze-guards, replacement of serving utensils, and food service by crew. Group 2 ships provided meals in a sitting service and not in a buffet line. Both groups applied the same rules about handwashing, maximum number of persons in food service areas, and distancing of tables and chairs. #Persons were considered fully vaccinated 14 d after the last dose of a COVID-19 vaccine. **Other measures applied in the community: gathering restrictions with maximum capacities, masking and physical distancing in indoor public spaces (theaters, gyms), hybrid policies for education and workplace settings, and proof of vaccination or negative tests to attend events (*2*). ††During the study period, the cumulative vaccine uptake (%) in the total population in EUMS/EEA (group 3) was ≈66% for the primary course (*3*).

We calculated incidence rate ratios, standardized incidence ratios (SIRs), and 95% CI using the epiR package in R (*5*). We used Fisher’s exact test to determine statistical significance. We considered p<0.05 statistically significant. We calculated SIRs for groups 1 and 2 by using epidemiologic COVID-19 data in EUMS and EEA countries during the study period as a reference population to calculate expected number of cases onboard (*4*) (Appendix).

The group 1 health measures protocol was reviewed and agreed upon by the Hellenic Ministry of Health’s national COVID-19 taskforce. The study received approval from the University of Thessaly’s Research Ethics Committee (protocol no. 103/16.11317 1.2021; decision no. 103/01.12.2021). Written consent for serologic testing was obtained from all crew members.

The risk for COVID-19 infection in group 2 (masked passengers of 9 ships) was 14.58 (95% CI 7.799–28.361) times lower than risk for group 1 (unmasked passengers) and 19.61 (95% CI 18.86–34.48) times lower than in group 3 (EUMS community members). Infection risk for unmasked passengers in group 1 was lower than in the community (SIR 0.744, 95% CI 0.512–1.045; p = 0.094) (Appendix).

## Conclusions

Our ecologic study demonstrated that COVID-19 infection risk among masked cruise ship passengers was 19.61 times lower than in the community (95% CI 18.86–34.48); the risk for infection among unmasked passengers was lower than in the community but not statistically significant (SIR 0.744, 95% CI 0.512–1.045; p = 0.094). Those findings suggest that NPMs implemented onboard the cruise ships were effective in reducing risk (*1*). Recent vaccination for the circulating variant appeared to contribute to reduced infection risk onboard ships, where vaccination coverage was almost 100%, compared with 66% cumulative vaccine uptake among the EUMS population (*3*). No outbreak occurred during the study period (group 1: median no. cases per voyage 1.00, range 0–15; group 2: median 0 cases per voyage, range 0–4). Of 44 close contacts of SARS-CoV-2–positive persons, 10 tested positive during quarantine, which could be attributed to protective effects of up-to-date vaccination for the circulating SARS-CoV-2 Delta variant. No deaths or severe cases were reported among the 11 cruise ships, despite the highly pathogenic nature of the Delta variant and older average age of cruise passengers.

Experimental studies in confined spaces demonstrated that masking is one of the most effective NPMs to prevent aerosol infection transmission (*6*). However, a systematic review of clinical trials in community settings and healthcare facilities demonstrated that wearing masks in the community likely makes little difference to outcomes compared with not wearing a mask (*7*). Masking in different settings (ships, hospitals, communities) might have different effects, however, the effectiveness of masking measures is likely influenced by how strictly those measures are enforced. During the pandemic, an absence of mask-wearing measures resulted in large outbreaks onboard ships (*8*,*9*). Our study demonstrated reduced COVID-19 incidence rates because of the protective effect of masking onboard ships. We suggest integrating use of high-filtration masks into routine case management, outbreak response measures, and preparedness and contingency planning for future public health emergencies of international concern. Crew members presented a lower infection risk than passengers and community populations, possibly because of mandatory mask use, recent vaccination, the strict enforcement of masking and vaccination policies, and reinforced education on symptoms and reporting requirements.

The first limitation of our study is that direct, individual observation of passenger and crew compliance was impossible in the uncontrolled environments of live cruises. The estimated case underreporting rates applied (1:4) were based on US data (February 2020–September 2021), but our study was implemented in Europe (July–November 2021), so differences could apply (*10*). The practice of 14-day quarantine and monitoring for disembarking passengers was applied only for close contacts of SARS-CoV-2–positive persons, so secondary cases could have been unidentified. We did not collect data on vaccination type, cabin occupancy, shore-based excursions, and onboard activities for the entire study population, so incidence rate differences for those factors could not be tested. Previous research of a COVID-19 cruise outbreak demonstrated that involvement in certain group activities (e.g., shows) and shore-based bus excursions were associated with infection, as well as a consistent dose-response relationship between number of cabinmates and attack rates in which attack rates decreased as passenger occupancy per cabin decreased (*11*,*12*). Alternative exposures, such as preembarkation queuing, social activities, contaminated surface contact, and common area use, deserve attention. Incubating passengers might not have been identified, but daily fever screening and diagnostic testing before boarding, during voyage, and before disembarking enhanced surveillance, reducing the possibility of undetected incubating COVID-19 cases (*1*). Strategies guaranteeing study protocol adherence were unfeasible on active voyages; however, enforcing company protocols and competent authority inspections maintained the intervention’s fidelity. Use of buffet lines in group 1 might be a confounder, but both groups applied identical food service occupancy limits; fomite transmission was unlikely given strict hand hygiene measures, replacement of serving utensils, sneeze-guards, and food service by crew. The ship company uniformly applied and enforced clear policies in groups 1 and 2. That uniform application was impossible in group 3 (communities) because implementation policies varied: full or partial; national, regional, or local; mandatory or voluntary; and groups targeted (i.e., at-risk persons, healthcare workers, travelers). Topics for further research include cost-effectiveness of NPMs on cruise ships in the context of pandemics, public health emergencies of international concern or during respiratory illness outbreaks. 

In conclusion, our ecologic study demonstrated the safe restart of cruise ship sector operations and indicated that mask use added an extra layer of protection; further studies should be conducted to verify the results. Masking should be considered in future public health emergencies when making decisions regarding NPMs and other measures that could interfere with international traffic and trade.

Appendix Additional information about nonpharmaceutical measures to prevent COVID-19 aboard cruise ships.
