# Supplementary material for: Interventional Study of Nonpharmaceutical Measures to Prevent COVID-19 Aboard Cruise Ships
Source: Emerg Infect Dis. 2024 May;30(5):1030–3. doi: 10.3201/eid3005.231364 (PMC11060450; doi:10.3201/eid3005.231364)
Supplement: Appendix — Additional information about nonpharmaceutical measures to prevent COVID-19 aboard cruise ships. [file 23-1364-Techapp-s1.pdf]

Article DOI: https://doi.org/10.3201/eid3005.231364

EID cannot ensure accessibility for supplementary materials supplied by authors. Readers who have difficulty accessing supplementary content should contact the authors for assistance.

Interventional Study of Nonpharmaceutical Measures to Prevent COVID-19 Aboard Cruise Ships

Appendix

Definitions and Methods

EU Healthy Gateways guidelines allowed medical mask or properly fitting respirator (FFP2) and, if unavailable, a non-medical "community" mask. When crew members or passengers were outside of their individual cabins, they had to use a facemask, exceptions included during eating and drinking. All crew members wore KN95/FFP2 facemasks (or equivalent) during the study period onboard Group 1 and 2 cruise ships. Passengers in Group 2 wore a medical mask or properly fitting respiratory protection (KN95/FFP2); however, if elevated numbers of COVID-19 cases were detected onboard, then all passengers were also provided with KN95/FFP2 (or equivalent) masks by the shipping company.

Recommendations for facemask use in communities and at points of entry (ports and airports) during the study period were as follows: ECDC recommended face mask use and physical distancing be continued when indoors (*1*). The US Centers for Disease Control and Prevention (CDC) advised that face mask use and physical distancing measures can be relaxed in indoor and outdoor settings applying to persons who are fully vaccinated if national/local regulations allow, but these measures should still be practiced by unvaccinated persons (*2*). At ports and airports during the study period, ECDC and EU HEALTHY GATEWAYS joint action recommended face mask use and physical distancing be continued when indoors (*1*,*3*). The use of face masks was mandated by the CDC at points of entry (e.g., airports) and on conveyances (aircrafts, rail, bus and public transportation) traveling inside, out of or into the U.S., with

exceptions for outdoor settings on conveyances. For any unvaccinated travelers, it was advised to maintain face mask use and physical distancing during travel (*2*).

Pre-embarkation questionnaires were administered by cruise line staff. Specific information collected included: demographic information (name, date/time of itinerary, port of disembarkation, cabin number, contact telephone number for 14 d after disembarkation), health questions regarding the past 14 d (presence of COVID-19 compatible symptoms, close contact of COVID-19 case, if provided care for COVID-19 case, if have been in close proximity or traveled on conveyance or shared household with a COVID-19 case).

A close contact of a COVID-19 case was defined as any person who had contact with a COVID-19 case within a timeframe ranging from 48 h before the onset of symptoms, of the case, or date of collection of a positive COVID-19 sample for an asymptomatic case, to 10 d after the onset of symptoms or date of collection of positive sample if asymptomatic. A: If a single or more cases sharing the same cabin have been identified onboard, then the following definitions of contacts should be applied: High-risk exposure (close) contact: A person who has stayed in the same cabin with a COVID-19 case. A person who had direct contact with infectious secretions of a COVID-19 case (e.g., being coughed on). A crew member who entered the cabin of a case while they were inside the cabin, without wearing appropriate PPE. For example, a crew member who cleaned the cabin of a case or who delivered food to the cabin. A person who has had face-to-face contact (on-board or on-shore) within 1.5 m for more than 15 min or who was in a closed environment for more than 15 min with a case. For passengers this could include, but is not limited to, participating in common activities, attending a class or sharing the same social space such as at a restaurant. This also includes contact with intimate partners. For crew this may include working in the same area as a case or socializing with a case (including fellow crew members), waiting on a table where a case was dining or leading a social activity where the case was participating. Healthcare workers or other persons providing direct care for a case without wearing appropriate PPE. Low-risk exposure (casual) contact: Risk assessment of individual cases and their contacts will be conducted by the ship's medical staff and/or public health authorities to identify the low-risk exposure (casual) contacts. Any data available from contact tracing technologies should also be considered. B. If three or more confirmed cases who are staying in two or more different cabins and who are not traveling together (excluding the cases identified the day of embarkation): Risk assessment of individual cases and their contacts

will be conducted by the public health authorities and the ship as part of contact tracing. Risk assessments could identify additional contacts who are not under the categories listed in part "A" of the definition. Any data available from contact tracing technologies will also be considered. Local/national regulations, definitions and procedures could also apply as part of the contact tracing.

Cruise ship A and B crew members were tested with SARS-CoV-2 IgG II assay (Abbott, Illinois, USA) which is a chemiluminescent microparticle immunoassay (CMIA) for the semiquantitative measurement of IgG antibodies that target the receptor binding domain (RBD) of the S1 subunit of the spike (S) protein of SARS-CoV-2. In addition, for cruise ship B serum samples were analyzed for the qualitative detection of IgG antibodies with the same method, (CMIA), using the ABBOTT SARS-CoV-2 IgG assay (Abbott, Illinois, USA), that targets the nucleocapsid (N) virus protein.

The shipping company provided education and training for crew members in Groups 1 and 2 when joining the ship, and then regularly via refresher trainings. Content included recognizing COVID-19 signs and symptoms, procedures and importance of reporting symptoms and appropriate implementation of NPMs. Information strategies were applied onboard for passengers' education. Both Group 1 and 2 cruise ships applied the same restrictions about the maximum capacities of passengers and crew onboard, as well as the same restrictions for maximum occupancy of specific ship spaces (e.g., dining rooms, theaters). The maximum capacity per cabin was two crew members. The number of passengers was restricted to a maximum of four per cabin during the study period. Furthermore, the overall population allowed to travel on cruise ships was restricted. Group 1 and 2 cruise ships applied the same rules for the dining room setting regarding distancing of tables and chairs. During the study period, NPMs were incorporated into the shipping company policy, and were a condition for crew members to work and passengers to voyage. NPMs policy was fully enforced by the companies for both crew members and passengers through security staff, while for crew members there were penalties, and if non-compliant they were asked to disembark. Both passengers and crew members were instructed on proper use of facemasks by security staff. Shows, films in the cinema and other social events for passengers were allowed onboard, with precautions to prevent overcrowding by limiting numbers of participants.

Certain measures were recommended to be in place during port visits, shore-based activities and excursions including rigorous hand hygiene, use of facemasks, health screening and contactless temperature measurement upon re-boarding the ship and measures to reduce overcrowding and maintain appropriate physical distancing during embarkation/disembarkation and ashore. Shore/excursions staff were recommended to be trained in procedures if a possible COVID-19 case was identified. While traveling in groups it was to be ensured that passenger groups maintained physical distancing from other tour groups and that disembarking and embarking travellers (from different ships or from the same ship but different voyages) did not occupy the same enclosed or semi-enclosed areas (e.g., gangways, terminal waiting spaces, check-in areas) at the same time.

The type of RADT used could be any type of RADT listed in the document "Common list of COVID-19 rapid antigen tests, including those of which their test results are mutually recognized, and a common standardized set of data to be included in COVID-19 test result certificates" (*4*). All ships in both groups 1 and 2 used the same types of RADT. The standards for RADT selection that were used in the community (Group 3) were the same as the cruise ships, since all EU MS had agreed on the common list for RADT. In Group 1 cruise ships, specimens that tested positive with RADT were sent for reverse transcription polymerase chain reaction r (RT-PCR) analysis and for Next Generation Whole Genome Sequencing.

References

1. European Centre for Disease Prevention and Control. Data on the daily number of new reported COVID-19 cases and deaths by EU/EEA country [cited 2022 Jul 23]. https://www.ecdc.europa.eu/en/publications-data/data-daily-new-cases-covid-19-eueea-country

2. Centers for Disease Control and Prevention. Interim public health recommendations for fully vaccinated people 2021 [cited 2021 Jun 29]. https://www.cdc.gov/coronavirus/2019-ncov/vaccines/fully-vaccinated-guidance.html

3. EU Healthy Gateways Joint Action. Advice for restarting cruise ship operations after lifting restrictive measures in response to the COVID-19 pandemic (version 2—April 2021) [cited 2021 Jun 29]. https://www.healthygateways.eu/Novel-coronavirus

4. European Commission Directorate-General for Health and Food Safety. EU health preparedness: EU common list of COVID-19 rapid antigen tests [cited 2024 Feb 9]. https://health.ec.europa.eu/system/files/2023-12/covid-19_eu-common-list-antigen-tests_en.pdf

5. Centers for Disease Control and Prevention. Estimated COVID-19 burden 2023 [cited 2024 Feb 9]. https://www.cdc.gov/coronavirus/2019-ncov/cases-updates/burden.html

Appendix Table 1. Results of statistical analysis among comparison Groups 1 and 2 for COVID-19 data, incidence rates and standardized incidence ratios using EUMS/EEA communities as a reference population*

Population and voyage characteristic	Group 1			Group 2, 9 cruise ships
	Cruise ship A	Cruise ship B	Total	
No. voyages	17	5	22	58
Median voyage duration, d (min, max)	7.00 (7.00, 14.0)†	7.00 (6.00, 12.0)†	7.00 (6.00, 14.0)	7.00 (3.00, 11.0)†
Total person-days (min, max)	96,194 (4,459, 11,312)	66,359 (9,486, 21,924)	162,553 (4,459, 21,924)	881,811 (5,379, 19,866)
Total passenger-days (min, max)	42,427 (1,561, 4,914)	32,428 (4,416, 11,676)	74,855 (1,561, 11,676)	529,029 (3,540, 13,419)
Total crew-days (min, max)	53,767 (2,884, 6,398)	33,903 (5,070, 10,248)	87,670 (2,884, 10,248)	352,740 (1,764, 12,768)
Median no. travelers, passengers and crew (min, max)	755 (637, 890)	1581 (1531, 1827)	786 (637, 1827)	2,097.5 (1,331, 2,838)
Median no. crew (min, max)	420 (412, 457)	845 (842, 854)	423.5 (412, 854)	875 (524, 1824)
Median no. passengers (min, max)	341 (223, 459)	736 (684, 973)	350.5 (223, 973)	1,204.5 (513, 1917)
Median percentage of vaccinated travelers onboard, crew and passengers (min, max)‡	100 (99.72, 100)	100 (100, 100)	100 (99.72, 100)	100 (NA, NA)
Median percentage vaccinated crew (min, max)	100 (99.27, 100)	100 (76.25, 100)	100 (76.25, 100)	92.57 (41.61, 100)
Median percentage vaccinated passengers (min, max)	100 (100, 100)	100 (100, 100)	100 (100, 100)	100 (100, 100)
SARS-CoV-2 Spike IgG–positive, (<50 AU/mL)/ total tested (%)	434/436 (99.50)	225/225 (100.00)	659/661 (99.70)	NT
Antinucleocapsid IgG–positive (>1.40 index)/ total tested (%)	Not tested	36/225 (16)	36/225 (16)	NT
SARS-CoV-2–positive by RT-PCR (next generation whole genome sequencing result)	21/27 (9 Delta variant [AY lineages])			NT
Epidemiologic rates				
Total no. cases	31§	5¶	36	21#
Cases among crew (%)	3 (9.68)	0 (0)	3 (8.33)	5 (23.81)
Cases among passengers (%)	28 (90.32)	5 (100)	33 (91.67)	16 (76.19)
Median no. cases (min, max)	1.00 (0, 15.0)**	1.00 (0, 4.00)	1.00 (0, 15.0)	0 (0, 4.00)
Median no. cases in crew (min, max)	0 (0, 2.00)	0 (0, 0)	0 (0, 2.00)	0 (0, 2.00)
Median no. cases in passengers (min, max)	1.00 (0, 13.0)	1.00 (0, 4.00)	1.00 (0, 13.0)	0 (0, 4.00)
Total no.voyages with ≥1 case (%)	12 (70.59)	2 (40)	14 (63.64)	13 (22.41)
Incidence rate (95% CI)	0.322 (0.219–0.457)	0.075 (0.024–0.176)	0.221 (0.155–0.307)	0.024 (0.015–0.036)
Passenger incidence rate (95% CI)	0.660 (0.439–0.954)	0.154 (0.050–0.360)	0.441 (0.303–0.619)	0.030 (0.017–0.049)
Crew incidence rate (95% CI)	0.056 (0.012–0.163)	0 (0.000–0.110)	0.034 (0.007–0.100)	0.014 (0.005–0.033)

Population and voyage characteristic	Group 1			Group 2, 9 cruise ships
	Cruise ship A	Cruise ship B	Total	
Passenger SIR (observed/expected cases) (95% CI)††	–	–	0.744 (33/44) (0.512–1.045)	0.051 (16/313) (0.029–0.083)
Crew SIR (observed/expected cases) (95% CI)††	–	–	0.058 (3/52) (0.012–0.169)	0.024 (5/209) (0.008–0.056)
Characteristics of cases				
Fully vaccinated cases (%)	31 (100.00)	7 (100.00)	38 (100.00)	13 (61.90)
Symptomatic at time of diagnosis (%)	20 (64.51)	1 (14.29)	21 (55.26)	5 (23.81)
Symptomatic after diagnosis (%)	NA	1 (14.29)	1 (2.63)	4 (19.05)
Day case was detected				
Day of embarkation testing (%)‡‡	0	2 (28.57)	2 (5.26)	5 (23.81)
Mid-cruise/before disembarkation testing (%)	20 (64.52)	4 (80.00)	24 (63.16)	6 (28.57)
Tested after showing symptoms (%)	10 (32.26)	1 (20.00)	11 (28.95)	1 (4.76)
Crew routine 7-d testing (%)	1 (3.23)	0	1 (2.63)	5 (23.81)
Crew initial testing (%)	0	0	0	0
Close contact testing (%)	0	0	0	0
Close contacts				
Close contacts identified (crew)	3	14	17	24
Close contacts identified (passengers)	21	6	27	36
Mean no. close contacts per case (min, max)	0.797 (0, 1.0)	3.875 (3.75, 4.00)	1.308 (0, 4.0)	2.85 (0, 10.0)
Close contacts quarantined ashore (crew)	0	0	0	7
Close contacts quarantined ashore (passengers)	1	3	4	23
Close contacts that became positive (crew)	0	0	0	0
Close contacts that became positive during quarantine (passengers)	0	0	0	10

*NT, not tested; SIR, standardized incidence ratio; –, not applicable.
†Cruise ship A: 16 voyages had 7-d duration, 1 voyage had 15-d duration. Cruise ship B: 1 voyage had 6-d duration, 2 voyages had 7-d duration, 1 voyage had 8-d duration, and 1 voyage had 14-d duration. Group 2: 1 voyage had 3-d duration, 1 voyage had 4-d duration, 2 voyages had 6-d duration, 48 voyages had 7-d duration, 3 voyages had 10-d duration, and 3 voyages had 11-d duration.
‡During the study period, all passengers and crew members were required to be vaccinated as a condition to board cruise ships.
§1 voyage with 15 cases, 1 voyage with 3 cases, 3 voyages with 2 cases, and 7 voyages with 1 case.
¶1 voyage with 4 cases, 1 cruise with 1 case.
#1 voyage with 4 cases, 1 voyage with 3 cases, 3 voyages with 2 cases, and 8 voyages with 1 case.
**In response to the voyage with an elevation in case counts (n = 15), face masking by all passengers onboard during the voyage was required as a response measure.
††Reference population: COVID-19 incidence rate in European Union Member States/European Economic Area countries during the study period multiplied by 4 to reflect the actual incidence rate (assuming that 1 in 4 cases were reported).
‡‡Two cases in cruise ship B and 5 cases in Group 2 cruise ships were diagnosed on day of embarkation testing. These persons were not allowed to embark, and therefore were not included in the analysis.

Appendix Table 2. Incidence rate ratios (Group 1 vs Group 2) for passengers

Starting month of voyage	Number of cases		Time at risk, d		Incidence rate, cases/1,000-person days (95% CI)		Incidence rate ratio (95% CI)	p value
	Group 1	Group 2	Group 1	Group 2	Group 1	Group 2		
July	2	4	10,402	100,437	0.192 (0.023–0.695)	0.040 (0.011–0.102)	4.828 (0.437–33.686)	0.102
August	8	8	25,302	221,986	0.316 (0.137–0.623)	0.036 (0.016–0.071)	8.773 (2.870–26.817)	<0.001
September–October	23	4	40,957	218,646	0.562 (0.356–0.843)	0.018 (0.005–0.047)	30.696 (10.488–122.109)	<0.001
July–October	33	16	74,855	529,029	0.441 (0.303–0.619)	0.030 (0.017–0.049)	14.576 (7.799–28.361)	<0.001

Appendix Table 3. Incidence rate ratios (Group 1 vs. Group 2) for crew members*

Starting month of voyage	Cases		Time at risk, d		Incidence rate (95% CI)†		Incidence rate ratio (95% CI)	p value
	Group 1	Group 2	Group 1	Group 2	Group 1	Group 2		
July	0	2	14,630	112,252	0.000 (0.000–0.252)	0.018 (0.002–0.064)	NaN	0.999
August	1	3	32,363	141,674	0.031 (0.001–0.172)	0.021 (0.004–0.062)	1.459 (0.028–18.174)	0.561
September–October	2	0	43,589	117,826	0.046 (0.006–0.166)	0.000 (0.000–0.031)	NaN	0.073
July–October	3	5	87,670	352,740	0.034 (0.007–0.100)	0.014 (0.005–0.033)	2.414 (0.375–12.408)	0.201

*NaN, not a number (indicating number of cases was 0 in 1 of 2 groups).
†Incident rate = cases/1,000-person days.

Appendix Table 4. Standardized Incidence Ratios using the European Union Member States/ European Economic area countries as the reference population*

Population group	Observed cases	Expected cases	Standardized incidence ratio (95% CI)	p value
Group 1 passengers	33	44.344	0.744 (0.512–1.045)	0.094
Group 1 crew members	3	51.936	0.058 (0.012–0.169)	<0.001
Group 2 passengers	16	313.399	0.051 (0.029–0.083)	<0.001
Group 2 crew members	5	208.965	0.024 (0.008–0.056)	<0.001
Groups 1 and 2	57	618.68	0.092 (0.067–0.119)	<0.001

*During the study period, 8,455,007 COVID-19 cases were reported in EUMS/EEA countries. The EUMS/EEA countries' total population was 453,090,377 and the study duration was 126 d, which corresponds to 57,089,387,502 person-days. Thus, incidence in the EUMS/EEA community population for the study period was 0.148 per 1,000 person-days. COVID-19 case detection methods in the cruise ship populations (Groups 1 and 2) were intensified through regular, documented RADT screening conducted by healthcare staff, which did not take place in the community (Group 3). Therefore, undetected and underreporting of COVID-19 cases in the community population was expected. To overcome underestimation of COVID-19 incidence rates in the community due to undetected or unreported cases, the number of reported COVID-19 cases in the community was multiplied by four, as the US Centers for Disease Control and Prevention estimated that from February 2020–September 2021, one in four COVID–19 infections were reported (95% uncertainty interval 3.4–4.7) (*5*). In our study, SIR was calculated using 33,820,028 as the total number of COVID-19 cases in EUMS/EEA countries (estimated community incidence rate: 0.592 per 1,000). EUMS/EEA, European Union Member States/European Economic Area; RADT, rapid antigen detection test.

Appendix Table 5. Type of vaccines for crew members for group 1

Type of Vaccine	Ship A no. crew members (%), n = 437	Ship B no. crew members (%), n = 864
Pfizer	382 (87.4)	39 (4.5)
AstraZeneca	37 (8.5)	314 (36.3)
Johnson & Johnson	6 (1.4)	371 (42.9)
Sinopharm	3 (0.7)	1 (0.1)
Moderna	2 (0.5)	13 (1.5)
CoronaVac (SinoVar)	3 (0.7)	124 (14.5)
Pfizer AZ	4 (0.8)	2 (0.2)
